# Coupling Ion Specificity of the Flagellar Stator Proteins MotA1/MotB1 of *Paenibacillus* sp. TCA20

**DOI:** 10.3390/biom10071078

**Published:** 2020-07-20

**Authors:** Sakura Onoe, Myu Yoshida, Naoya Terahara, Yoshiyuki Sowa

**Affiliations:** 1 Department of Frontier Bioscience, Research Center for Micro-Nano Technology, Hosei University, 3-7-2 Kajino-cho, Koganei, Tokyo 184-8584, Japan; sakura.onoe@protein.osaka-u.ac.jp (S.O.); miyu.yoshida.54@hosei.ac.jp (M.Y.); 2 Department of Physics, Chuo University, 1-13-27 Kasuga, Bunkyo-ku, Tokyo 112-8551, Japan; terahara.19r@g.chuo-u.ac.jp

**Keywords:** flagellar motor, coupling ion, divalent cation

## Abstract

The bacterial flagellar motor is a reversible rotary molecular nanomachine, which couples ion flux across the cytoplasmic membrane to torque generation. It comprises a rotor and multiple stator complexes, and each stator complex functions as an ion channel and determines the ion specificity of the motor. Although coupling ions for the motor rotation were presumed to be only monovalent cations, such as H^+^ and Na^+^, the stator complex MotA1/MotB1 of *Paenibacillus* sp. TCA20 (MotA1^TCA^/MotB1^TCA^) was reported to use divalent cations as coupling ions, such as Ca^2+^ and Mg^2+^. In this study, we initially aimed to measure the motor torque generated by MotA1^TCA^/MotB1^TCA^ under the control of divalent cation motive force; however, we identified that the coupling ion of MotA1^TCA^MotB1^TCA^ is very likely to be a monovalent ion. We engineered a series of functional chimeric stator proteins between MotB1^TCA^ and *Escherichia coli* MotB. *E. coli* Δ*motAB* cells expressing MotA1^TCA^ and the chimeric MotB presented significant motility in the absence of divalent cations. Moreover, we confirmed that MotA1^TCA^/MotB1^TCA^ in *Bacillus subtilis* Δ*motAB*Δ*motPS* cells generates torque without divalent cations. Based on two independent experimental results, we conclude that the MotA1^TCA^/MotB1^TCA^ complex directly converts the energy released from monovalent cation flux to motor rotation.

## 1. Introduction

Most swimming bacteria can swim towards their favorable environments by rotating their helical flagella [1,2]. Each flagellum is rotated by a rotary molecular motor embedded in the bacterial cell envelope at its base [3]. The rotation of the bacterial flagellar motor is driven by the flux of the coupling ions across the cytoplasmic membrane. The motor comprises a rotor and multiple stator units. Each stator unit contains two types of membrane proteins called the Mot complex and functions to conduct the coupling ions via a channel in order to generate mechanical torque. Previous studies reported that various Mot complexes work in a wide range of bacterial species, such as MotA/MotB in H^+^-driven motors of *Escherichia coli*, PomA/PomB in Na^+^-driven motors of *Vibrio alginolyticus*, MotP/MotS in Na^+^-driven motors of *Bacillus subtilis*, and MotP/MotS in Na^+^, K^+^, and Rb^+^-driven motors of *Bacillus alcalophilus* [4,5,6,7]. All these Mot complexes share a common structure and function; a peptidoglycan (PG) binding domain to anchor the stator unit to the PG layer, a transmembrane (TM) domain to transduce the coupling ion(s) across the membrane, and a large cytoplasmic domain to interact with the rotor proteins [8]. Therefore, several functional chimeric motors comprise various combinations of rotor and stator proteins [9,10,11,12,13]. One such representative chimera motor used for functional analysis, is the Na^+^-driven motor of *E. coli* that combines the rotor of the H^+^-driven *E. coli* motor with the chimeric stator PomA/PotB [11,14,15,16,17,18]. The PomA/PotB stator contains PomA from *V. alginolyticus* and a fusion protein, which replaces the PG binding domain of PomB from *V. alginolyticus* with that of MotB from *E. coli* for efficient anchoring to the PG layer of *E. coli* cells [19].

Recently, MotA1^TCA^/MotB1^TCA^ in the flagellar motor of *Paenibacillus* sp. TCA20, which was isolated from a Ca^2+^ rich hot spring in Japan, was reported to use Mg^2+^ and Ca^2+^ as coupling ions for its rotation rather than the monovalent cations, which were utilized by all other flagellar motors [20]. The MotA1^TCA^/MotB1^TCA^ can interact with the rotor of the *B. subtilis* motor and then generate a torque [20]. This novel type of flagellar motor is presumed to shed light on the mechanism of energy conversion of the motor, because the charge number (z) contributes to the gained energy from the ion flux. In the bacterial flagellar motor, the free energy from a single ion passage across the cytoplasmic membrane comprises an electrical component (z*e*V_m_) and a chemical component (*k_B_T* ln (C_i_/C_o_)), where *e* is the elementary charge, V_m_ is the transmembrane voltage, *k_B_* is Boltzmann’s constant, *T* is absolute temperature, and C_i_ and C_o_ are inside and outside concentrations of the coupling ion, respectively [3,21]. Therefore, the energy released from a single divalent ion moving down the electrochemical gradient is much larger than that of a single monovalent ion.

**Figure 1:**
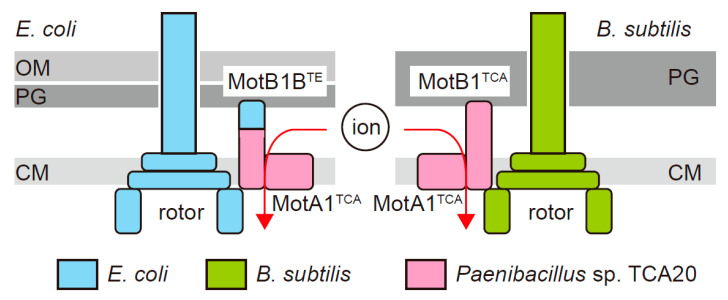
Schematic diagram of chimeric flagellar motors in *Escherichia coli* and *Bacillus subtilis*. In *E. coli*, the chimeric stator proteins, MotA1^TCA^/MotB1B^TE^, interact with an *E. coli* rotor. In *B. subtilis*, MotA1^TCA^/MotB1^TCA^ interacts with a *B. subtilis* rotor. The regions derived from *E. coli*, *B. subtilis*, and *Paenibacillus* sp. TCA20 are colored light blue, green, and magenta, respectively. OM: outer membrane, PG: peptidoglycan layer, CM: cytoplasmic membrane.

In the present study, we aimed to demonstrate the energy conversion efficiency of the electrochemical potential of divalent ions on the flagellar motor to understand the mechanism of converting chemical energy into mechanical rotational work by the ion passage. We initially replaced the PG binding domain of MotA1^TCA^/MotB1^TCA^ with that of *E. coli* MotB (MotB^EC^), and analyzed the function of a series of chimera stator proteins in *E. coli* cells (Figure 1). Next, we measured the rotations of single motors driven by MotA1^TCA^/MotB1^TCA^ in *B. subtilis* cells (Figure 1). Surprisingly, our data revealed that MotA1^TCA^/MotB1^TCA^ couples monovalent ions, presumably protons, for its rotation, rather than divalent ions.

## 2. Materials and Methods

### 2.1. Strains, Plasmids, Growth Conditions, and Media

*E. coli* strains and plasmids used in this study are listed in Tables S1 and S2, respectively. For motility assay, plasmids were transformed into RP6665 to restore motility. For tethered cell assays, plasmids were transformed into JHC36 [15]. The *E. coli* cells were grown in T-broth (1% Bacto Tryptone, 0.5% sodium chloride) at 30 °C. Ampicillin was added at 50 µg/mL to preserve the plasmids. Inducer arabinose was added to the growth medium at 1 mM.

The *B. subtilis* strains used in this study are listed in Table S1. The *B. subtilis* cells were grown in Spizizen I medium plus 1 mM MgCl_2_ and 0.6 mM IPTG at 37 °C [20]. Spizizen I medium (pH 8.0) contained 10% Spizizen salts, 0.5% glucose, 0.02% casamino acids, 0.1% yeast extract, 10 μg/mL tryptophan, and10 μg/mL lysine. Spizizen salts contained 85 mM K_2_HPO_4_, 40 mM KH_2_PO_4_, 15 mM (NH_4_)_2_SO_4_, 6 mM sodium citrate, and 0.8 mM MgSO_4_.

### 2.2. Construction of Plasmids Encoding Chimera Stator Proteins

The primers used for plasmid construction are listed in Table S3. To construct a series of chimera stator proteins between MotB1^TCA^ and MotB^Ec^, in vivo *E. coli* cloning (iVEC) was carried out following the recently published method [22]. To clone *motA1^TCA^motB1^TCA^* into pBAD24 by iVEC method, DNA fragments amplified by PCR using *Paenibacillus* sp. TCA20 genome as a template with primers 1200 and 1201 and using pBAD24 as a template with primers 1198 and 1204 were cotransformed to ME9783 strain, yielding pSHU157. To obtain pSHU161, PCR products from RP437 genome were used as a template with primers 1313 and 1316, and PCR products from pSHU157 were used as a template with primers 1317 and 1318 which were fused by iVEC method. pSHU162–pSHU167 were constructed with the same procedures used for pSHU161 via different primer combinations.

### 2.3. Strain Construction of B. subtilis

To construct the *B. subtilis* strain expressing MotA1^TCA^MotB1^TCA^ and sticky flagellar filaments, we followed the procedures reported as the rotation measurement of the *B. subtilis* flagellar motor [23] with minor modifications. Firstly, we removed two BamHI sites in *motA1^TCA^motB1^TCA^*. Silent mutations were introduced by site-directed mutagenesis by PCR using pSHU157 as a template with primers 1228 and 1229, and primers 1230 and 1231, yielding pSHU1347. Secondly, we added *motA1^TCA^motB1^TCA^* under an IPTG-inducible P*_grac_* promoter. *motA1^TCA^motB1^TCA^* was amplified by PCR using pSHU1347 as a template with 1222 and 1223. The PCR product was digested with BamHI and SmaI and was then cloned into the BamHI and SmaI site of pHT01, yielding pSHU1348. Thirdly, we subcloned P*_grac_*-*motA1^TCA^motB1^TCA^* into the pDR67 integration vector. P*_grac_*-*motA1^TCA^motB1^TCA^* was amplified by PCR using pSHU1348 as a template with primers 1224 and 1225. The PCR product was digested with SmaI and SphI and then cloned into the SmaI and SphI sites of pDR-hagsticky, yielding pSHU1351. Eventually, P*_grac_*-*motA1^TCA^motB1^TCA^* and P*_hag_*-hagsticky were introduced to ΔABΔPSΔHag by selecting a chloramphenicol-resistant and amylase-negative phenotype, yielding SHU399.

### 2.4. Motility Assays

Motility of *E. coli* cells expressing the chimera stator proteins was tested by the motility plate and swimming assays. Semi-solid agar plates (0.25% Bacto Agar and 1 mM arabinose in T-broth) were inoculated from single colonies and incubated at 30 °C for 9 h. The cells were allowed to swim in 10 mM potassium phosphate buffer (pH 7.0) containing 1 mM EDTA or 1 mM MgCl_2_. Cell suspensions with an optical density of around 0.8 at 600 nm were collected by centrifugation at 3000× *g* for 3 min, and washed thrice with the observation buffer. The speed of swimming cells was measured by tracking the cells using a custom-made program written in LabVIEW, after capturing their images at 60 fps by CMOS camera for 10 s.

### 2.5. Rotation Measurement by Tethered Cell Assays

To reduce the number of flagellar filaments extended from the cell body, the sticky flagellar filaments of the cultured cells were sheared by being passed through either a Pasteur pipette or a syringe with a 26G needle. Cells were harvested by centrifugation at 3000× *g* for 3 min and the pellet was resuspended in either phosphate buffer or HEPES-Tris buffer. This process was repeated thrice. Cells were incubated in a sample chamber for 15 min to attach on the coverslips via their sticky filament. After flushing the unbound cells with the same buffer, spinning cells, which attach on the coverslips via single filaments, were captured at 60 fps by CMOS camera for 10 s [24]. To observe the effect of the electrochemical potential of Mg^2+^ on the motor speed, the buffer in a sample chamber was exchanged by running the buffer containing appropriate concentrations of EDTA, MgCl_2_, or CCCP (carbonyl cyanide m-chlorophenyl hydrazone). The rotational speed was determined by trajectory analysis of the cell body using a custom-made program written in LabVIEW.

## 3. Results

### 3.1. MotA1^TCA^ and Chimeras between MotB1^TCA^ and MotB^Ec^ Function as A Stator in E. coli

**Figure 2:**
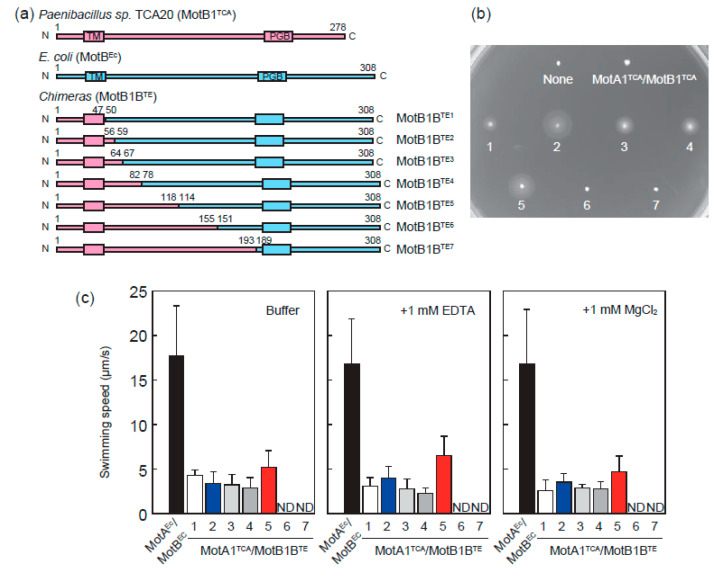
Characterization of chimeric stator in *E. coli*. (**a**) Design of a series of domain swap chimeras MotB1B^TE^. The region derived from MotB1^TCA^ and MotB^Ec^ are colored light magenta and blue, respectively. TM and PGB represent the transmembrane and peptidoglycan binding domains, respectively. Numbers above each diagram indicate the amino acid sequence numbers. Depending on the swapping sites, chimera proteins MotB1B^TE^ are numbered from 1–7. (**b**) Motility of *E. coli* cells expressing chimera stator proteins on semi-solid agar plate. The numbers correspond to chimeras in (**a**). (**c**) Swimming speed of *E. coli* cells expressing chimera stator proteins in phosphate buffer (left) containing 1 mM EDTA (center) and 1 mM MgCl_2_ (right). ND represents that no swimming cells were detected.

Most quantitative functional assays of the flagellar motor have been established in *E. coli* cells; however, MotA1^TCA^/MotB1^TCA^ was reported to be nonfunctional as a stator in *E. coli* cells [20]. Therefore, we constructed a series of chimeras between MotB1^TCA^ and MotB^Ec^, following the design of PomA/PotB in previous work (Figure 2a) [11]. MotB protein has a single TM domain with an ion-binding site and a large PG binding domain. All constructed chimeric proteins contain TM domain derived from MotB1^TCA^ and PG binding domain derived from MotB^Ec^. The sites for swapping were chosen by comparing the sequence similarities between MotB1^TCA^ and MotB^Ec^, and a series of chimeras were named as MotB1B^TE1^–MotB1B^TE7^.

To examine whether the constructed chimeric Mot complexes were able to rotate the flagellar rotor of *E. coli*, we transformed a series of plasmids encoding MotA1^TCA^ and MotB1B^TE^ to *E. coli* Δ*motAB* cells and checked their motility on semi-solid agar plates. We found that chimeras except MotA1^TCA^/MotB1B^TE6^ and MotA1^TCA^/MotB1B^TE7^ can restore the motility of *E. coli* Δ*motAB* cells (Figure 2b).

We measured the swimming speed of the *E. coli* Δ*motAB* cells expressing chimera stator proteins in phosphate buffer (Figure 2c, left panel). The swimming speed of *E. coli* Δ*motAB* cells expressing chimeras MotA1^TCA^/MotB1B^TE1^–MotB1B^TE5^ was ~4 μm/s, which is considerably slower than the cells expressing wild type MotA^Ec^/MotB^Ec^. A similar analysis was performed in the presence of 1 mM MgCl_2_ (Figure 2c, right panel). An appropriate concentration was used for the swimming motility of *Paenibacillus* cells; however, no significant effect of Mg^2+^ was observed on the motility of *E. coli* Δ*motAB* cells expressing chimeras MotA1^TCA^/MotB1B^TE1^–MotB1B^TE5^. Moreover, the motility was maintained despite Mg^2+^-chelation by EDTA (Figure 2c, center panel). Therefore, presumably Mg^2+^ is not used as a coupling ion for motor rotation.

### 3.2. Chimeric Motor in E. coli Is Driven by Protons Rather than Divalent Ions

**Figure 3:**
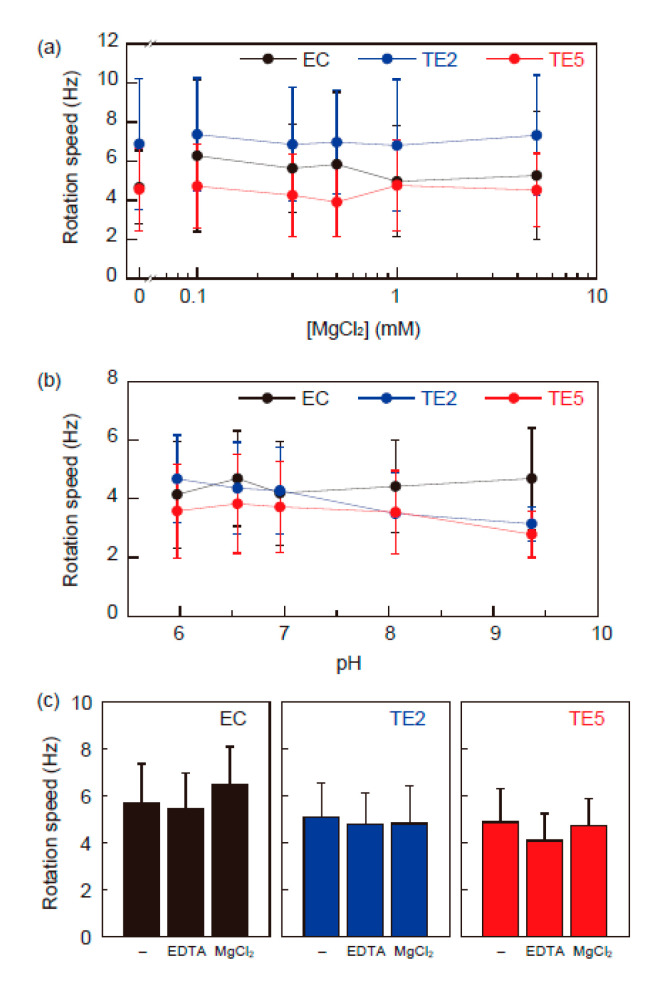
Rotational speed of single motors driven by chimera stators in *E. coli*. (**a**) Effect of Mg^2+^ concentrations on the motor speed. Buffer contains 10 mM potassium phosphate (pH 7.0) and indicated MgCl_2_. In the absence of MgCl_2_, 0.1 mM EDTA was added to the buffer. (**b**) Effect of pH on the motor speed. 10 mM potassium phosphate buffer containing 10 mM EDTA was adjusted to the desired pH by titration with HCl or KOH. (**c**) Motor speeds of EC (left), TE2 (center), and TE5 (right) in HEPES-Tris buffer. EDTA and MgCl_2_ indicate the buffers containing 10 mM EDTA and 10 mM MgCl_2_, respectively.

We further investigated the function of single motors driven by MotA1^TCA^/MotB1B^TE2^ and MotA1^TCA^/MotB1B^TE5^, which efficiently support cell motility on semi-solid agar. We transformed the plasmids encoding MotA^Ec^/MotB^Ec^, MotA1^TCA^/MotB1B^TE2^, and MotA1^TCA^/MotB1B^TE5^ into *E. coli* Δ*motAB* expressing sticky flagellar filament, namely EC, TE2, and TE5. Cells were tethered to a glass surface spontaneously via a single sticky filament, and their rotation was captured and analyzed. Figure 3a illustrates the dependence of Mg^2+^ concentrations on the motor speed of EC, TE2, and TE5, wherein the motor speeds of TE2 and TE5 were similar to that of EC at up to 5 mM MgCl_2_, and were independent of the Mg^2+^ concentration. Figure 3b depicts the dependence of pH on the motor speed of EC, TE2, and TE5, wherein the motor speed of EC was constant in the range of pH 6–9, as previously reported [25]. The motors of TE2 and TE5 also rotated at an approximately constant speed despite the pH conditions. Since the buffer contains 10 mM EDTA-2K to chelate the divalent ions completely, the potential cations driving these motors are limited to be protons or potassium ions.

To test whether the motors of TE2 and TE5 require the potassium ions for rotation, we measured the motor rotation in 100 mM HEPES-Tris buffer (pH 7.0). The motors of EC, TE2, and TE5 rotated at a similar speed to that of the potassium phosphate buffer, even in the absence of potassium ion (Figure 3c). Addition of 10 mM EDTA or 10 mM MgCl_2_ had no effect on these motor speeds. These results suggested that protons are very likely used as an energy source for motor rotation of TE2 and TE5 [21,26].

### 3.3. MotA1^TCA^/MotB1^TCA^ Couples Monovalent Cations in ΔmotABΔmotPS B. subtilis

**Figure 4:**
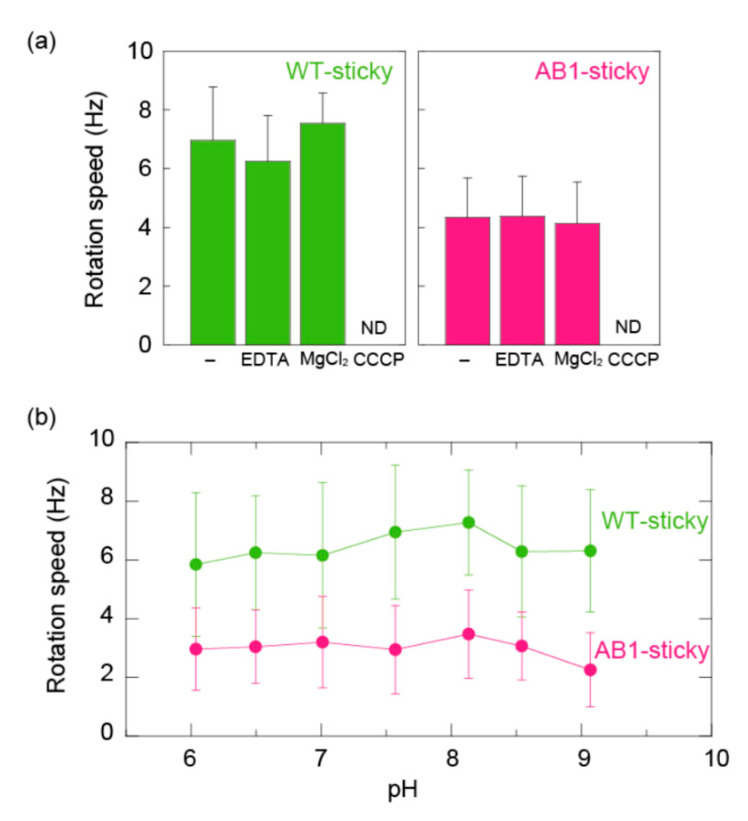
Rotational speed of single motors driven by MotA1^TCA^/MotB1^TCA^ in *B. subtilis*. (**a**) Motor speeds of WT-sticky (left) and AB1-sticky (right) in 10 mM potassium phosphate buffer containing 0.1 mM EDTA. EDTA, MgCl_2_, and CCCP indicate buffers containing 10 mM EDTA, 10 mM MgCl_2_, and 10 mM MgCl_2_ and 25 μM CCCP, respectively. ND represents that no spinning tethered cells were detected. (**b**) Effect of pH on the motor speed. 10 mM potassium phosphate buffer containing 10 mM EDTA was adjusted to the desired pH by titration with HCl or KOH.

MotA1^TCA^/MotB1^TCA^ was reported to interact with a rotor of *B. subtilis* and support the cell motility of *B. subtilis* Δ*motAB*Δ*motPS* cells. We found that AB1 cells (Δ*motAB*Δ*motPS* cells expressing MotA1^TCA^/MotB1^TCA^) swam very slowly (less than 2 μm/s) while wobbling, even in the buffer containing 10 mM MgCl_2_, meaning that swimming analysis of AB1 cells would not be suitable for further detailed analysis. Therefore, we investigated the function of MotA1^TCA^/MotB1^TCA^ by a single motor assay in *B. subtilis* using the system of Hag-sticky filament, which was recently developed [23]. We initially constructed the strain expressing MotA1^TCA^/MotB1^TCA^ and Hag-sticky by following the procedures as previously reported [23], and named it as AB1-sticky. Moreover, we measured the rotational speed of WT-sticky, which expresses a wild-type flagellar motor and Hag-sticky as a control. The rotation speed of tethered AB1-sticky cells was about half that of WT-sticky cells in 10 mM potassium phosphate buffer (pH 8.0) containing 0.1 mM EDTA (Figure 4a). As in *E. coli* cells, the addition of 10 mM EDTA or 10 mM MgCl_2_ had no effect on these motor speeds. Collapsing protonmotive force by the addition of 25 μM CCCP completely hindered the motor rotation of both WT-sticky and AB1-sticky. Furthermore, the rotational speeds of WT-sticky and AB1-sticky were constant within error in the range of pH 6–9 as well as in *E. coli* cells (Figure 4b). Therefore, we concluded that MotA1^TCA^/MotB1^TCA^ couples monovalent ions for its rotation.

## 4. Discussion

Imazawa et al. reported that the flagellar motor of *Paenibacillus* sp. TCA20 is driven by the flux of divalent ions and MotA1^TCA^/MotB1^TCA^, which acts as a stator in the motor, couples the divalent ion flow to motor rotation [20]. This stator complex was also reported to interact with the rotors of *B. subtilis* and to use divalent ions for torque generation without requiring protonmotive force. In the present study, to investigate of energy conversion of MotA1^TCA^/MotB1^TCA^, we performed single motor assays in the *E. coli* and *B. subtilis* flagellar system, respectively. A stator complex of MotA1^TCA^ and chimera MotB1B^TE1–5^ generated torque by interacting with a rotor of the *E. coli* flagellar motor. The chimeras with high MotB1^TCA^ occupancy, such as MotB1B^TE6^ and MotB1B^TE7^, did not work in *E. coli*, presumably by not forming a structure to anchor to the PG layer of *E. coli* cells [19]. Our data indicated that the chimera stator complexes use monovalent ions, presumably protons, rather than divalent cations for torque generation, although they possess TM domain of MotA1^TCA^/MotB1^TCA^, which is a core of energy converter (Figure 3a,b). MotA1^TCA^/MotB1^TCA^ functions as a stator in the stator-less *B. subtilis* cells, but does not require divalent cations for torque generation, which is not concordant to the previous report (Figure 4a). Some bacteria, such as *Bacillus clausii*, were reported to switch the ion specificity of their motor depending on the environmental pH [27]. Therefore, we checked the possibility of the ion specificity switching of chimeras in *E. coli* and MotA1^TCA^/MotB1^TCA^ in *B. subtilis*; however, all stator complexes function in the absence of divalent cations in the range of pH 6–9. Collectively, the results from this study revealed that MotA1^TCA^/MotB1^TCA^ does not directly couple the divalent ion to motor rotation, thus challenging the results of the previous report.

Although the reason for discrepancy between the results of this study and the previous reports remains unclear, one possible hypothesis that can be considered is the difference of measuring the flagellar rotation. Bacterial swimming is caused by the integration of complex processes, such as the flagellar motor rotation, motor switching related to chemotaxis, and bundle formation of flagella. [28,29]. If the divalent cations affect the chemotactic signaling and increase the switching frequency of the motor without changing the swimming speed, then the swimming speed may reduce or swimming may stop, leading to pseudodependence of divalent cations on motor function. Especially when analyzing slow swimming motions, the effects of fluctuations due to fluid flow and Brownian motion of cells must be carefully considered. In the present study, we measured the rotational speeds of single motors which is assumed to be the most reliable and robust assessment for the motor function itself. Alternatively, divalent cations might have a positive effect on the folding of stator complexes. As recently discovered in MotP/MotS of *B. subtilis*, binding of Na^+^ to MotS induces the structural transition of its PG binding domain for anchoring to the PG layer [30]. If divalent cations activate the folding of the PG binding domain of MotB1^TCA^ to interact with a rotor, the fraction of stator complexes generating torque would increase its dependence on divalent cation concentrations. Here, MotA1^TCA^/MotB1^TCA^ was overexpressed using a P*_lac_* promotor without LacI; therefore, some portion of stator complexes might fold stochastically without requiring divalent cations and assemble in a motor to generate torque. This possibility would be tested by analyzing the effect of the expression level of MotA1^TCA^/MotB1^TCA^ on the motor speed in the absence of divalent ions. Nevertheless, future studies are warranted.

Our experiments revealed that MotA1^TCA^/MotB1^TCA^ in *B. subtilis* cells couples monovalent ions, presumably protons, to its rotation. To the best of our knowledge, the coupling ion specificity of the intact stator complex does not change depending on the expressed species; therefore, we speculate that the flagellar motor of *Paenibacillus* sp. TCA20 directly uses the proton flux for its energy source [11]. Nevertheless, the possibility that MotA1^TCA^/MotB1^TCA^ uses divalent cations in extreme conditions, particularly the environment where *Paenibacillus* sp. TCA20 was isolated, cannot be ruled out completely. Considering this case, single motor assay would be suitable for revealing the motor properties.

## Supplementary Materials

The following are available online at https://www.mdpi.com/2218-273X/10/7/1078/s1, Table S1: Strains used in this study, Table S2: Plasmids used in this study, Table S3: Primers used in this study.
