# Supplementary material for: Coupling Ion Specificity of the Flagellar Stator Proteins MotA1/MotB1 of Paenibacillus sp. TCA20
Source: Biomolecules. 2020 Jul 20;10(7):1078. doi: 10.3390/biom10071078 (PMC7407149; doi:10.3390/biom10071078)
Supplement: Supplementary file 1 [file biomolecules-10-01078-s001.pdf]

Supplementary Materials

Coupling Ion Specificity of the Flagellar Stator Proteins MotA1/MotB1 of *Paenibacillus* sp. TCA20

Sakura Onoe, Myu Yoshida, Naoya Terahara and Yoshiyuki Sowa

Table S1. Strains used in this study

Strain	Relevant characteristics	Source or Reference
E. coli		
RP437	wild type for motility	Parkinson JS
RP6665	Δ*motAB*	Parkinson JS
JHC36	Δ*cheY* Δ*pilA* Δ*motAB fliC*-*sticky*	Inoue et al. 2008 [13]
ME9783	strain for iVEC	NRBP
B. subtilis		
AB1	Δ*motAB* Δ*motPS lacA::*P_{xylA}*-motAB1*	Imazawa et al. 2016 [20]
ΔABΔPSΔHag	Δ*motAB* Δ*motPS* Δ*hag*::*spec*	Terahara et al. 2017 [19]
WT-sticky	Δ*hag::spec amyE*::P_{hag}-*hagsticky*	Terahara et al. 2017 [19]
SHU399 (AB1-sticky)	Δ*motAB* Δ*motPS* Δ*hag*::*spec* *amyE*::P_{grac}-*motAB*1, P_{hag}-*hagsticky*	This study

Table S2. Plasmids used in this study

Plasmid	Relevant characteristics	Antibiotic resistance	Source or Reference
pBAD24	expression vector by P_{ara} promoter	Amp^r	Lab stock
pYS16	pBAD24-*motA*^{Ec}*motB*^{Ec}	Amp^r	This study
pSHU157	pBAD24-*motA1*^{TCA}*motB1*^{TCA}	Amp^r	This study
pSHU161	pBAD24-*motA1*^{TCA}*motB1B*$^{TE-3}$	Amp^r	This study
pSHU162	pBAD24-*motA1*^{TCA}*motB1B*$^{TE-2}$	Amp^r	This study
pSHU163	pBAD24-*motA1*^{TCA}*motB1B*$^{TE-1}$	Amp^r	This study
pSHU164	pBAD24-*motA1*^{TCA}*motB1B*$^{TE-4}$	Amp^r	This study
pSHU165	pBAD24-*motA1*^{TCA}*motB1B*$^{TE-5}$	Amp^r	This study
pSHU166	pBAD24-*motA1*^{TCA}*motB1B*$^{TE-6}$	Amp^r	This study
pSHU167	pBAD24-*motA1*^{TCA}*motB1B*$^{TE-7}$	Amp^r	This study
pHT-AB	pHT01 + P_{grac}-*motAB* of *B. subtilis*	Amp^r Cm^r	Terahara et al. 2017 [19]
pDR-hagsticky	pDR67 + P_{hag}-*hagsticky*	Amp^r Cm^r	Terahara et al. 2017 [19]
pSHU1347	pBAD24-*motA1*^{TCA}(R55R, D174D) *motB1*^{TCA}	Amp^r	This study
pSHU1348	pHT01 + P_{grac}-*motA1*^{TCA}*motB1*^{TCA}	Amp^r Cm^r	This study
pSHU1351	pDR-hagsticky + P_{grac}-*motA1*^{TCA}*motB1*^{TCA}	Amp^r Cm^r	This study

Table S3. Primers used in this study

Primer	Sequence
1198_pBAD24-f	cggatgagagaagattttca
1200_motA1^{TCA}-f	gggcttgcaggaggaattcaatggatatcgcgaccctaat
1201_motB1^{TCA}-r	tgaaaatcttctctcatccgttactcactttgcagctgtc
1204_pBAD24-r	tgaattcctcctgcaagccc
1222_BamHI-motA1^{TCA}-f	accgggatccatggatatcgcgaccctaatcgg
1223_motB1^{TCA}-XmaI-r	tccccccgggttactcactttgcagctgtcgcag
1224_XmaI-Pgrac-f	tccccccgggtttaaatgcaaccgtttttc
1225_motB1^{TCA}-SphI-r	acatgcatgcttactcactttgcagctgtcgcag
1228_motA1^{TCA}(R55R)-f	ctatccaatgcaccgcatccgcactctgcct
1229_motA1^{TCA}(R55R)-r	aggcagagtgcggatgcggtgcattggatag
1230_motA1^{TCA}(D174D)-f	tggccatctgacggacccgagtcagctcggt
1231_motA1^{TCA}(D174D)-r	accgagctgactcgggtccgtcagatggcca
1313_motB^{Ec}-f for TE3	cttcaaactacctttgcgactgcggttacgggcgg
1314_motB^{Ec}-f for TE2	aagtatgaggaagtcgcggagtacttccggactcc
1315_motB^{Ec}-f for TE1	tatgcgatgagccggtccagcccaaaagagctgat
1316_motB^{Ec}-r for TE1-7	atcttctctcatccgtcacctcggttcggctgatg
1317_pBAD24-f for TE1-7	gccgaaccgaggtgacggatgagagaagattttca
1318_motB1^{TCA}-r for TE3	cgtaaccgcagtcgcaaaggtagtttgaagggagc
1319_motB1^{TCA}-r for TE2	ccggaagtactccgcgacttcctcatacttgcttg
1320_motB1^{TCA}-r for TE1	ctcttttgggctggaccggctcatcgcatacatca
1331_motB^{Ec}-f for TE4	attgactacccctctaatagtgaaagcccaattcc
1332_motB^{Ec}-f for TE5	accgaacgagagtctcgattgcggaaattgcgggg
1333_motB^{Ec}-f for TE6	gacaagcctcaagggcagatcatcgatagccagaa
1334_motB^{Ec}-f for TE7	agggatctcgagacacgtattagcctttcaggtca
1335_motB1^{TCA}-r for TE4	tgggctttcactattagaggggtagtcaatgacac
1336_motB1^{TCA}-r for TE5	caatttccgcaatcgagactctcgttcggtcaaag
1337_motB1^{TCA}-r for TE6	gctatcgatgatctgcccttgaggcttgtccacta
1338_motB1^{TCA}-r for TE7	tgaaaggctaatacgtgtctcgagatccctaaaca
